# The rib index is not practically affected by the distance between the radiation source and the examined child

**DOI:** 10.1186/1748-7161-10-S2-S8

**Published:** 2015-02-11

**Authors:** Theodoros B Grivas, Konstantinos C Soultanis, Christina Mazioti, Vasileios Kechagias, Antonios Akriotis, Konstantinos Athanasopoulos, Christos Naskas

**Affiliations:** 1 Department of Orthopaedics and Traumatology, “Tzaneio” General Hospital, Tzani and Afendouli 1, Piraeus 18536, Greece; 2 1st Department of Orthopaedics, University of Athens, University General Hospital “Attikon”, 1 Rimini str, Chaidari, Athens 12462, Greece

## Abstract

**Background:**

All lateral spinal radiographs in idiopathic scoliosis (IS) show a Double Rib Contour Sign (DRCS) of the thoracic cage, a radiographic expression of the rib hump. The outline of the convex overlies the contour of the concave ribs. The rib index (RI) method was extracted from the DRCS to evaluate rib hump deformity in IS patients. The RI was calculated by the ratio of spine distances d1/d2 where d1 is the distance between the most extended point of the most extending rib contour and the posterior margin of the corresponding vertebra on the lateral scoliosis films, while d2 is the distance from the least projection rib contour and the posterior margin of the same vertebra, (Grivas et al 2002). In a symmetric thorax the “rib index” is 1.

This report is the validity study of DRCS, ie how the rib index is affected by the distance between the radiation source and the irradiated child.

**Methods:**

The American College of Radiology's (2009) guidelines for obtaining radiographs for scoliosis in children recommends for the scoliotic - films distance to be 1,80 meters.

Normal values used for the transverse diameter of the ribcage in children aged 6-12 years were those reported by Grivas in 1988.

**Results:**

Using the Euclidean geometry, it is shown that in a normal 12-year old child d1/d2 = 1.073 provided that the distance ΔZ ≈ 12cm (11,84) and EA = 180cm, with transverse ribcage diameter of the child 22 cm.

**Conclusions:**

This validity study demonstrates that the DRCS is substantially true and the RI is not practically affected by the distance between the radiation source and the irradiated child. The RI is valid and may be used to evaluate the effect of surgical or conservative treatment on the rib cage deformity (hump) in children with IS. It is noted that RI is a simple method and a safe reproducible way to assess the rib hump deformity based on lateral radiographs, without the need for any other special radiographs and exposure to additional radiation.

## Background

The rib index (RI) extracted from the double rib contour sign was introduced for the first time at the 25^th^ “Nicolas Giannestras - Panayiotis Smyrnis” Anniversary Symposium of Spinal Column Diseases, at the Porto Rio Hotel, Patras, Greece, 21-23 May 1999, [1]. It was subsequently presented at the International Research Society of Spinal Deformities meeting at Clermont Ferrand, Château du Marand, France, 23-26 May 2000, [2] and published two years later, [3]. This report is, on a geometrical model, a validity study of DRCS, ie how the RI is affected by the distance between the radiation source and the irradiated child.

## Methods

### 1. The child – film distance

When a AIS child is radiographically examined, the child - films distance must be standardized according to existing experts’ recommendations. The American College of Radiology's (2009) guidelines for obtaining radiographs for scoliosis in children recommends for the scoliotic - films distance to be 1,80 meters, [4].

### 2. The thoracic dimension values

**Figure 1:**
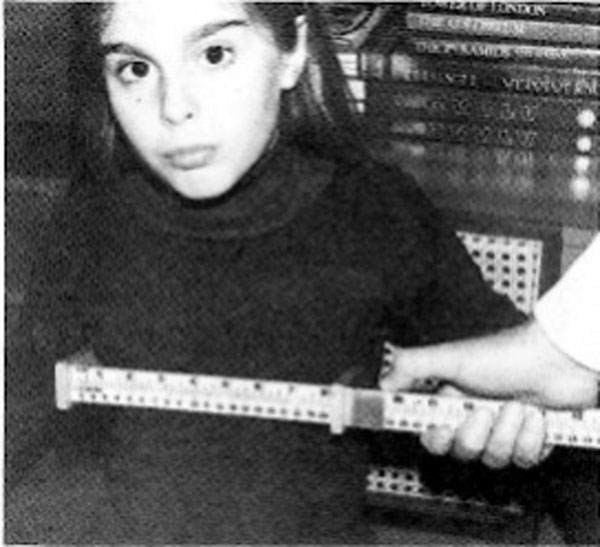
**The transverse ribcage diameter**. The way the transverse diameter of the ribcage is documented using a special anthropometric tool. This figure is the original one used in the author’s PhD Thesis, (http://www.didaktorika.gr/eadd/handle/10442/3440)

**Figure 2:**
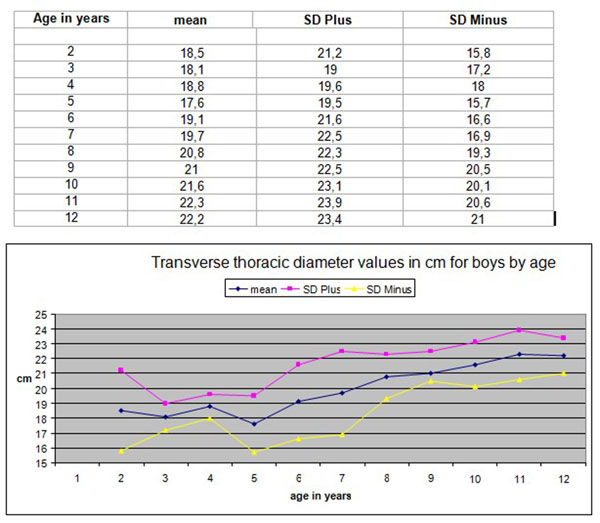
**Transverse thoracic diameter values in cm for boys by age.** The values of the transverse thoracic diameter clinical measurements in centimeters by age (2-12 years old) in boys.

**Figure 3:**
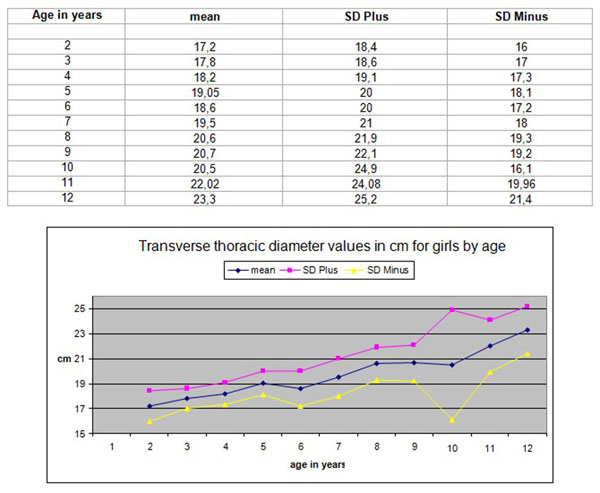
**Transverse thoracic diameter values in cm for girls by age.** The values of the transverse thoracic diameter clinical measurements in centimeters by age (2-12 years old) in girls.

Anthropometric data of children’s thoracic cage dimensions are rarely reported. The PhD thesis of the first author provided such data. Therefore normal values used for the transverse diameter of the ribcage in children aged 6-12 years were those reported by Grivas in 1988, [5]. These values are the readings of the clinical measurements of the ribcage at its maximum diameter using an anthropometric tool, Figure 1. The values of the transverse ribcage diameter clinical measurements are presented in centimetres by age (2-12 years old) in boys and girls, Figures 2 and 3.

### 3. Patient positioning for the radiographic examination

The position of the IS patient for the radiographic examination is very important. Each radiograph must be taken by technicians trained to do radiography in a timeless standardized and similar way, in any hospital or laboratory.

## Results

**Figure 4:**
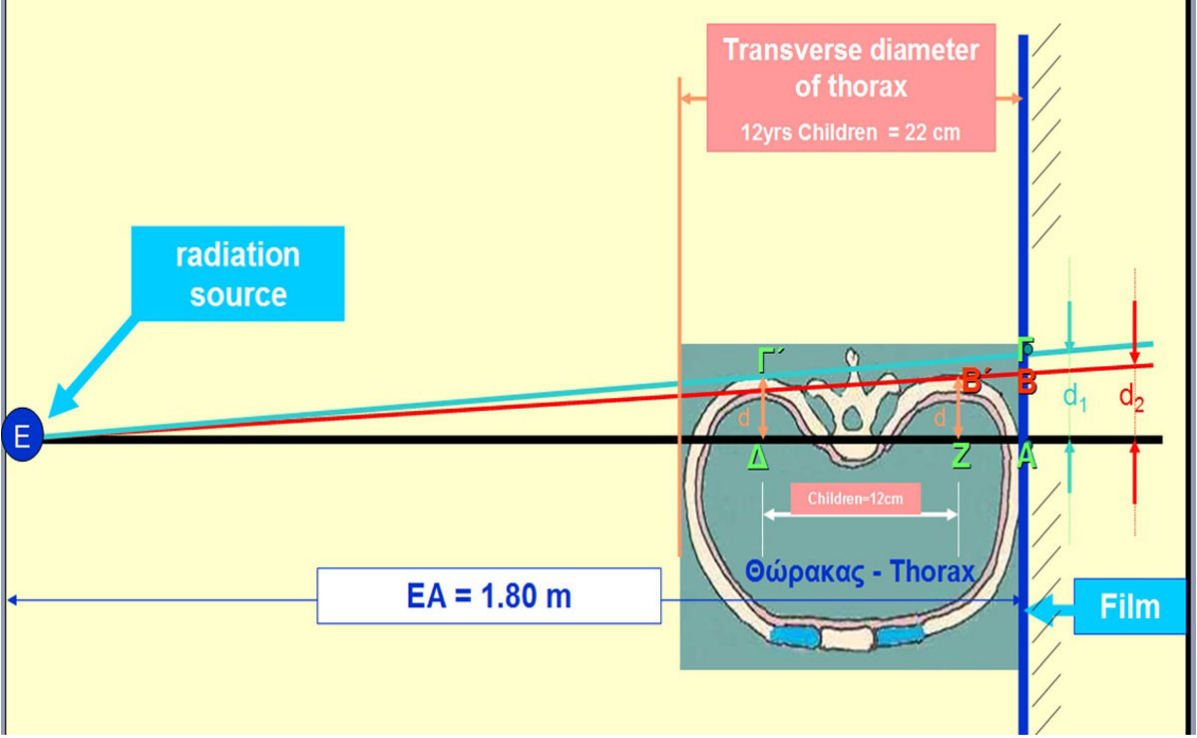
**Radiograph obtaining set up.** It is depicted the set up for obtaining the radiograph and the used points, (Greek letters), for the assessment of d1/d2 quotient. (The figure is not original and the original can be found in the abstract published in the Standard Supplement of the Sapporo 2014 IRSSD meeting, [11]).

**Figure 5:**
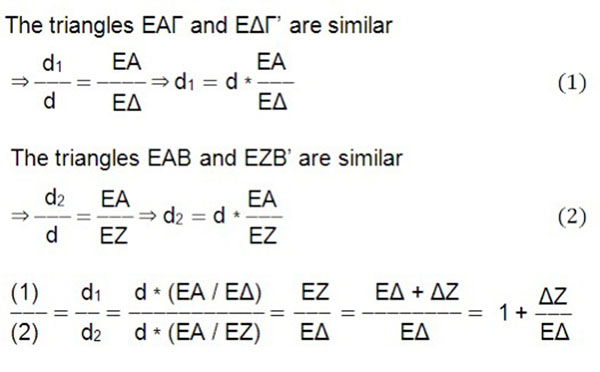
**Assessment of d1/d2 quotient.** The Euclidean geometry used for the assessment of d1/d2 quotient. (The figure is not original and the original can be found in the abstract published in the Standard Supplement of the Sapporo 2014 IRSSD meeting, [11]).

In a setting for obtaining the radiograph, Figure 4, using the Euclidean geometry, Figure 5, it is shown that d1/d2=1.073 in a normal 12-year old child, provided that ΔZ≈12cm (11,84) and EA = 180cm, with transverse ribcage diameter of the child 22 cm.

## Discussion

The RI is used to assess the transverse plane thoracic deformity, due to its reliability, [6] and simplicity, [7]. This index can be easily used not only to assess any brace effectiveness on the rib hump deformity correction, [8], but also to objectively document the changes of the rib hump deformity in a progressive AIS patient treated with the physiotherapeutic specific scoliosis exercise (PSSE) methods, [9]. The RI was used to assess the ribcage changes after operative treatment in AIS, [7,10], as well.

The rib hump expresses the transverse plane deformity of the thoracic cage. Therefore the RI method can be used to assess the deformity in this plane, a method which was missing so far from the every day praxis. The importance of using the RI method is that it is a simple, valid, reliable and safe reproducible way to assess the rib hump deformity based on lateral radiographs, without the need for any other special radiographs and exposure to additional radiation, [7].

Finally one additional benefit of this method is its implementation not only in prospective but also in retrospective studies, to assess the transverse plane deformity of the ribcage during treatment using the existing initially obtained spinal radiographs of IS patients, provided that the radiography is performed in any hospital or laboratory in a timeless standardized and similar way.

## Conclusions

This validity study demonstrates that the DRCS is not an artifact but is substantially true. The RI is not practically affected by the distance between the radiation source and the irradiated child.

## Competing interests

There are no competing interests to disclose.

## Authors' contributions

TBG: Conceived the idea of RI, drafted the text and searched the literature. All authors contributed their professional skills to the inclusions of the text.

All authors have read and approved the final manuscript.

This is the extended abstract of IRSSD 2014 program book [O8-5, page 72], [11].

## Declarations

Publication charges for this collection were funded by IRSSD 2014 Sapporo.

This article has been published as part of *Scoliosis* Volume 10 Supplement 2, 2015: Research into Spinal Deformities 9: Short Papers from the IRSSD 2014 Meeting. The full contents of the supplement are available online at http://www.scoliosisjournal.com/supplements/10/S2.
